# Deciphering the differential response of two human fibroblast cell lines following Chikungunya virus infection

**DOI:** 10.1186/1743-422X-9-213

**Published:** 2012-09-20

**Authors:** Vincent G Thon-Hon, Melanie Denizot, Ghislaine Li-Pat-Yuen, Claude Giry, Marie-Christine Jaffar-Bandjee, Philippe Gasque

**Affiliations:** 1 Immunopathology and Infection Research Grouping (IRG), EA4517, University of La Reunion, CHU Felix Guyon and CYROI, St Denis, La Reunion, France; 2 Microbiology/Virology Laboratory, CHU Felix Guyon of La Reunion, St Denis, La Reunion, France

**Keywords:** CHIKV, Type I IFN, HS 633T, HT-1080, RIG-I, TLR7, STAT-1

## Abstract

**Background:**

Chikungunya virus (CHIKV) is an arthritogenic member of the *Alphavirus* genus (family *Togaviridae*) transmitted by *Aedes* mosquitoes. CHIKV is now known to target non hematopoietic cells such as epithelial, endothelial cells, fibroblasts and to less extent monocytes/macrophages. The type I interferon (IFN) response is an early innate immune mechanism that protects cells against viral infection. Cells express different pattern recognition receptors (including TLR7 and RIG-I) to sense viruses and to induce production of type I IFNs which in turn will bind to their receptor. This should result in the phosphorylation and translocation of STAT molecules into the nucleus to promote the transcription of IFN-stimulated antiviral genes (ISGs). We herein tested the capacity of CHIKV clinical isolate to infect two different human fibroblast cell lines HS 633T and HT-1080 and we analyzed the resulting type I IFN innate immune response.

**Methods:**

Indirect immunofluorescence and quantitative RT-PCR were used to test for the susceptibility of both fibroblast cell lines to CHIKV.

**Results:**

Interestingly, the two fibroblast cell lines HS 633T and HT-1080 were differently susceptible to CHIKV infection and the former producing at least 30-fold higher viral load at 48 h post-infection (PI). We found that the expression of antiviral genes (RIG-I, IFN-β, ISG54 and ISG56) was more robust in the more susceptible cell line HS 633T at 48 h PI. Moreover, CHIKV was shown to similarly interfere with the nuclear translocation of pSTAT1 in both cell lines.

**Conclusion:**

Critically, CHIKV can control the IFN response by preventing the nuclear translocation of pSTAT1 in both fibroblast cell lines. Counter-intuitively, the relative resistance of HT-1080 cells to CHIKV infection could not be attributed to more robust innate IFN- and ISG-dependent antiviral responses. These cell lines may prove to be valuable models to screen for novel mechanisms mobilized differentially by fibroblasts to control CHIKV infection, replication and spreading from cell to cell.

## Background

Chikungunya virus (CHIKV) is an arthritogenic member of the *Alphavirus* genus (family *Togaviridae*) transmitted by *Aedes* mosquitoes [1]. CHIKV is responsible for a febrile illness called CHIK fever which has an incubation period usually comprised between 3 to 7 days (range, 2-12 days) [2,3]. The first cases of patients infected by CHIKV described acute onset of high fever (temperature usually above 38.9°C), severe joint pain, and rash as classic clinical symptoms [4]. CHIKV has been responsible for explosive outbreaks since 2005 in the Indian Ocean. CHIKV targets human non hematopoietic cells including fibroblasts, epithelial, neuronal and endothelial cells and to less extent hematopoietic cells (*e.g.* monocyte-derived macrophages and primary cultures of macrophages) [5-10]. CHIKV is an enveloped virus and its genome consists in a positive single-stranded RNA molecule of 11805 nucleotides long [11]. It is composed of two open reading frames (ORFs). The 5’ ORF encodes non-structural proteins (nsP1 to nsP4) which are multifunctional and form together the virus replicase. The 3’ ORF encodes the structural proteins (capsid [C], envelope glycoproteins [E1 and E2], E3 and 6 k) [12-14].

Induction of type I interferons (IFN-α and β) by intracellular sensors such as the Toll-like receptors (TLRs) located in endosomes (*e.g.* TLR7) and the cytosolic RIG-like receptors (RLRs) (*e.g.* RIG-I) represents an early innate immune response against viruses [15-18] TLR7 recognizes single-strand RNA [19-21] whereas RIG-I detects viral genomic RNA bearing 5’-triphosphates, single and double-strand RNAs (dsRNA) [22]. Interaction of RIG-I DExD/H box domain with viral dsRNA induces conformational changes which promotes downstream signaling cascade. The mitochondrial antiviral-signaling protein (MAVS, also known as VISA, Cardiff or IPS-1), an adaptator molecule, is then recruited and activates the tank binding kinase 1 (TBK1 also IKKε in lymphoid cells). TBK1 thus phosphorylates the interferon regulatory factor 3 (IRF-3) at specific serine residues [23,24]. Then IRF-3 dimerizes and translocates into the nucleus to promote the expression of IFN-α and β [25]. IFN response is initiated by the binding of type I IFNs to the cell surface IFN-α/β receptor (IFNAR) in an autocrine and paracrine manner [26,27]. IFNAR subsequently activates the Janus kinases proteins (Jak1 and Tyk2), which in turn phosphorylate signal transducers and activators of transcription 1 and 2 (STAT1 and STAT2) [28]. Phosphorylated STAT1 and STAT2 form heterodimers, migrate into the nucleus and associate with IRF-9 (also known as p48 or ISGF-3γ) to form a transcription factor complex termed IFN-stimulated gene factor 3 (ISGF-3) [26]. Active ISGF-3 interacts with a specific DNA sequence called the IFN-stimulated response element (ISRE) present in the promoter region of IFN-stimulated genes (ISGs) to promote ISG transcription. The expression of various ISGs is induced to clear viral infection, including the protein kinase R (PKR) which activates the shutdown of protein translation [29,30]. Recent investigation of innate immune reaction in human fibroblasts showed that CHIKV induces innate immune activation via the adaptor molecule IPS-1 in human fibroblasts [31]. In this work, we deciphered further the downstream innate immune response of two human fibroblast cell lines HS 633T and HT-1080 infected by CHIKV on the ground that they showed differential capacity to be infected and to replicate CHIKV.

## Results

### The human fibroblast cell lines HS 633T and HT-1080 are differently susceptible to CHIKV infection

**Figure 1:**
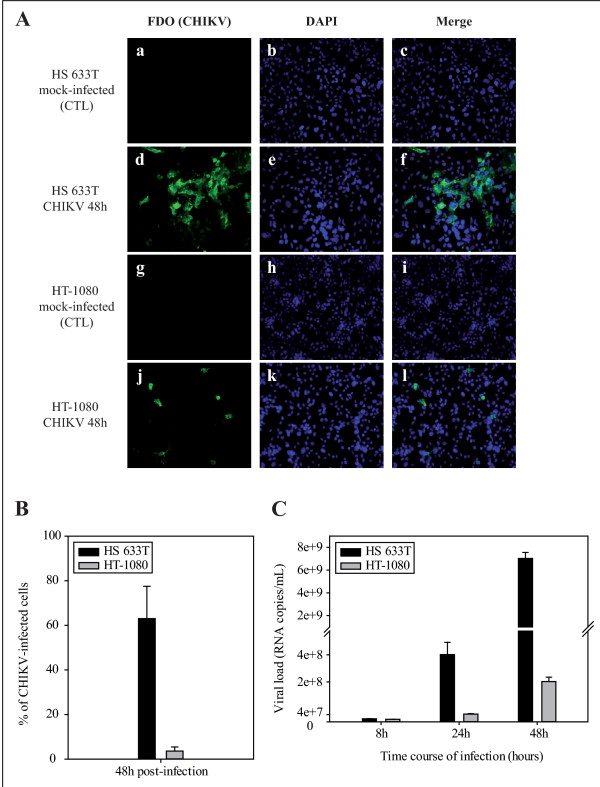
**Human fibroblast cell lines HS 633T and HT-1080 are differently susceptible to chikungunya virus (CHIKV) infection.** (**A**) Immunostaining (polyclonal anti-CHIKV, green) of HS 633T and HT-1080 cells infected with CHIKV MOI of 1 (d, e, f and j, k, l respectively) or mock-infected (**a, b, c** and **g, h, i** respectively). Nuclei were stained with DAPI (blue), view X200. The experiments were performed in triplicate. (**B**) Percentage of HS 633T and HT-1080 CHIKV-infected (green staining) at a MOI of 1 at 48 h post-infection. Green fluorescent cells were counted in three observation fields and results are expressed as mean ± standard error. (**C**) Quantification of the viral load by real time qRT-PCR from HS 633T and HT-1080 supernatants infected with CHIKV MOI 1 for 8, 24 and 48 h. The experiment was done in duplicate and results are expressed as mean ± standard error.

To evaluate their susceptibility to the virus, HS 633T and HT-1080 cells were grown on glass coverslips in 24 well plates and incubated for 48 h with a MOI of 1 of the clinical CHIKV isolate clone #4 [32]. In mock-infected cells (CTL), no CHIKV was detected by immunostaining (Figure 1Aa, c and g, i). At 48 h post infection (PI), 63.02% ± 14.48 of HS 633T cells were stained for CHIKV (Figure 1B). The infection spreads in larger clusters from the initially infected cells (Figure 1Ad, f). Surprisingly at the same time point, only 3.62% ± 1.83 of HT-1080 cells were infected by CHIKV (Figure 1B). The infection did not spread in clusters in HT-1080 cells since infected cells were found isolated at 48 h PI (Figure 1Aj, l). These results indicate that HS 633T are highly susceptible to CHIKV infection while HT-1080 cells are less susceptible. Next, we wanted to evaluate the ability of both fibroblast cell lines to produce viral progeny in the medium. Interestingly at 8 h post-infection, identical levels of CHIKV RNA copies were detected in the supernatant of both cell lines (1.68x10^7^ ± 1.23x10^6^ viral RNA copies/mL in HS 633T compared to 1.46x10^7^ ± 3.70x10^5^ in HT-1080) whereas at 24 and 48 h PI the number of viral RNA copies was clearly higher in HS 633T compared to HT-1080 cells (Figure 1C). For instance, 3.70x10^8^ ± 6.74x10^7^ viral RNA copies/mL were detected in HS633T versus 4.26x10^7^ ± 2.80x10^6^ in HT-1080 at 24 h PI. At 48 h PI the values reached 7.03x10^9^ ± 5.31x10^8^ in HS 633T and 2.22x10^8^ ± 2.45x10^7^ in HT-1080). 

### Robust HS 633T- and milder HT-1080- innate immune responses against CHIKV

**Figure 2:**
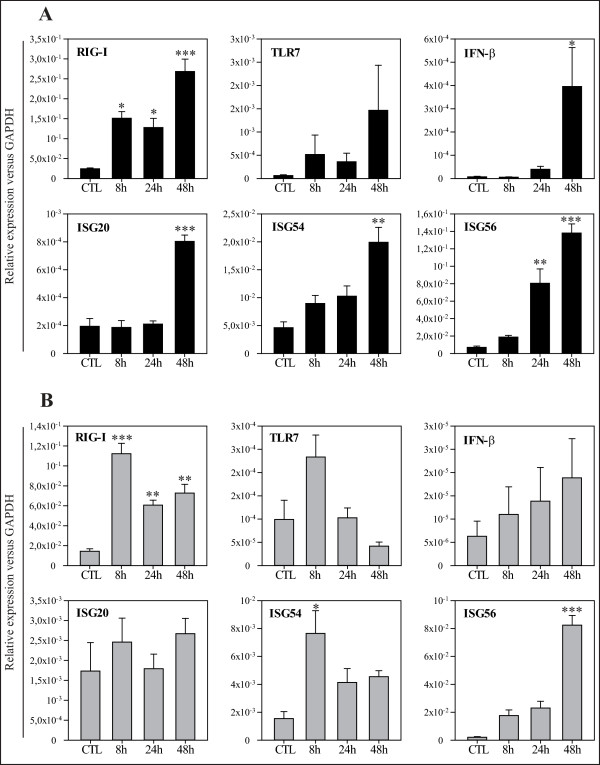
**CHIKV modulates the expression of the IFN pathway and IFN-stimulated genes in both human fibroblast cell lines HS 633T and HT-1080.** Relative expression of RIG-I, TLR7, IFN-β, ISG20, ISG54 and ISG56 from cells infected with CHIKV MOI of 1 for 8, 24, 48 h and mock-infected cell (CTL) in HS 633T (**A**) or HT-1080 (**B**) as assessed by real-time quantitative PCR. All experiments were done in triplicates and results are expressed as mean ± standard error (*: p < 0.05; **: p < 0.01; ***: p < 0.001).

We next investigated whether the expression of antiviral genes from the IFN pathway was differentially modulated by CHIKV in both fibroblast cell lines. We used GAPDH as a housekeeping gene and to compare the relative expression between both cell lines. First, we found that both cell lines expressed equally well the receptors involved in RNA virus sensing (RIG-I and TLR7), IFN-β and three of the main ISGs (ISG20, ISG54 and ISG56) in basal conditions (Figure 2A, B).

In response to CHIKV infection, the relative expression of RIG-I significantly increased in HS 633T at 8 h (1.51x10^-1^ ± 1.66x10^-2^, p < 0.05), 24 h (1.28x10^-1^ ± 2.29x10^-2^, p < 0.05) and 48 h PI (2.68x10^-1^ ± 3.12x10^-2^, p < 0.001) when compared to mock-infected cells (2.46x10^-2^ ± 2.02x10^-3^) (Figure 2A). The same modifications in RIG-I expression was noted in infected HT-1080 (Figure 2B). Interestingly, we found higher expression of RIG-I in infected HS 633T when compared to infected HT-1080 at 24 h (p < 0.05) and 48 h PI (p < 0.001). The relative expression of IPS-1 (data not shown) and TLR7 (Figure 2A, B) were not significantly affected in both cell lines following CHIKV infection.

The relative expression of IFN-β was increased significantly at 48 h PI in HS 633T (3.96x10^-4^ ± 1.67x10^-4^, p < 0.05) (Figure 2A) but was not affected in HT-1080 (Figure 2B). Moreover, the relative expression of IFN-β was significantly higher in HS 633T when compared to HT-1080 at 48 h PI (1.89x10^-5^ ± 8.43x10^-6^ for HT-1080, p < 0.01).

All three ISGs tested were significantly upregulated in HS 633T-infected cells at 48 h PI while more modest upregulation were observed in HT-1080-infected cells for ISG54 (8 h) and ISG56 (48 h) PI and not for ISG20. For instance, the relative expression of ISG54 was significantly higher in HS 633T than in HT-1080 at 24 h (1.03x10^-2^ ± 1.84x10^-3^ versus 4.14x10^-3^ ± 9.90x10^-4^, p < 0.05) and 48 h PI (1.99x10^-2^ ± 2.67x10^-3^ versus 4.55x10^-3^ ± 4.24x10^-4^, p < 0.001). Similarly, the relative expression of ISG56 was significantly higher in HS 633T than in HT-1080 at 24 h (8.04x10^-2^ ± 1.65x10^-2^ versus 2.31x10^-2^ ± 4.77x10^-3^, p < 0.001) and at 48 h PI (1.38x10^-1^ ± 1.05x10^-2^ versus 8.24x10^-2^ ± 6.90x10^-3^, p < 0.001).

These results suggest that the relative resistance of HT-1080 to be infected and replicate CHIKV cannot be attributed to higher expression of antiviral genes such as IFN-β and ISGs involved in the innate immune response.

### CHIKV interferes with the nuclear translocation of pSTAT1 in both HS 633T and HT-1080 cell lines

**Figure 3:**
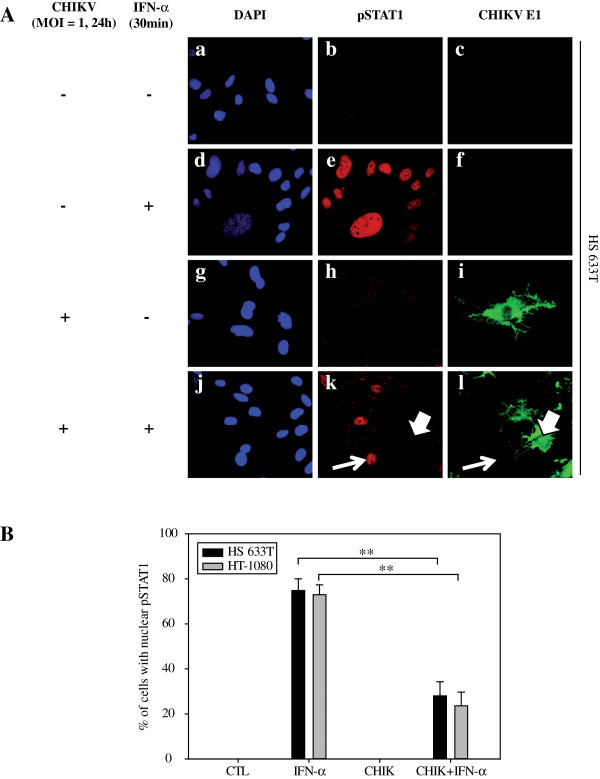
**CHIKV interferes equally with the nuclear translocation of phospho-STAT1 in both HS 633T and HT-1080 cells.** (**A**) Double immunostaining was performed for pSTAT1 (red) and CHIKV envelope E1 (green) using HS633T cells either mock-infected **(a, b, c)**; IFN-α-stimulated **(d, e, f)**; CHIKV-infected **(g, h, i)**; and finally CHIKV-infected and IFN-α-stimulated (j, k, l), view X600. Nuclei were stained in blue with DAPI. All experiments were done in triplicate. (**B**) Percentage of HS 633T or HT-1080 cells with nuclear pSTAT1 from experiments performed in A. Cells were counted in three random fields and the results are expressed as mean ± standard error (**: p < 0.01).

We next examined the capacity of CHIKV to control the nuclear translocation of phosphorylated STAT1 (pSTAT1) in both human fibroblast cell lines. As expected, pSTAT1 colocalized with DAPI staining indicating that pSTAT1 translocated into the nucleus of the large majority of HS 633T cells in response to exogenous IFN-α (Figure 3A: d, e). In CHIKV-infected HS 633T cells, CHIKV E1 was detected (Figure 3Ai) whereas pSTAT1 staining (nuclear and cytoplasmic stainings) could not be observed (Figure 3Ah). In CHIKV-infected and subsequently stimulated with IFN-α, less than 25% of cells were pSTAT1+ (Figure 3Ak, p < 0.01) when compared to 100% following IFN-α stimulation alone. Interestingly, we found that pSTAT1+ nuclei (long arrow) were present in CHIKV negative cells next to infected cells (Figure 3A; large arrow).

To test if CHIKV was capable of interfering with the nuclear translocation of pSTAT1 in both HS 633T and HT-1080, the number of cells stained for nuclear pSTAT1 was counted in either mock-infected (CTL); mock-infected and IFN-α stimulated (IFN-α); CHIKV-infected (CHIKV); and finally CHIKV-infected (MOI of 1, 24 h) and subsequently IFN-α stimulated (30 min). In IFN-α stimulated cells, robust levels of nuclear pSTAT1 were detected in both cell lines (HS 633T: 74.84% ± 5.18 and HT-1080: 72.98% ± 4.34). In CHIKV-infected and IFN-α stimulated cells, levels of nuclear pSTAT1 significantly decreased in both cell lines (HS 633T: 27.98% ± 6.33 and HT-1080: 23.61% ± 6.05, p < 0.01). Surprisingly, these results suggest that the difference of susceptibility to CHIKV infection between HS 633T and HT-1080 cells could not be attributed to a differential inhibition of nuclear pSTAT1 to mediate the antiviral response.

## Discussion

CHIKV is well known to infect both non hematopoietic and hematopoietic cells [5-10]. CHIKV not only infects macrophages but also human fibroblasts from cognitive tissues of different origins (skin, synovium) [5,10]. Couderc et al. demonstrated that in neonate mice with a mild infection, CHIKV primarily targets muscles, joints and skin fibroblasts, a cellular and tissue tropism similar to that reported in humans [5]. In a seminal study, Sourisseau and colleagues have shown that skin- and lung-derived fibroblasts (Hs 789.Sk and MRC5 respectively) were differentially infected by CHIKV [10] but through mechanisms ill-characterized.

In this paper we demonstrated that HS 633T cells behave as a susceptible fibroblast cell line to CHIKV infection in contrast to the other human fibroblast cell line HT-1080 where only a smaller percentage of cells were infected and released CHIKV progenies at lower levels. We hypothesized that this difference could be due to differential expression of TLR7 and/or RIG-I which are key sensors to control CHIKV infection at least in mouse embryonic fibroblasts [9]. Against our expectations, our results didn’t support this hypothesis and arguing for the role of additional antiviral mechanisms mobilized differentially by fibroblast cell lines.

The IFN system is a powerful antiviral mechanism capable of controlling most, if not all, virus infections in the absence of a functional adaptative immunity [28]. However, our analysis did not reveal a higher expression of IFN-β in HT-1080 and, in contrast, the expression was significantly more robust in HS 633T cells at 48 h PI probably as a consequence of higher levels of viral RNA within the cells. This result is consistent with a recent study on primary human foreskin fibroblasts (HFs) [31]. White and colleagues demonstrated that infection of HFs by CHIKV triggered the transcription of IFN-β at 24 h PI.

Viruses have also developed several strategies to control the downstream IFN response to replicate, persist and cause chronic diseases. Here, we showed that CHIKV clinical isolate interfered equally well with the nuclear translocation of activated STAT1 in HS 633T and HT-1080 fibroblast cell models even in the presence of exogenous recombinant IFN-α. This observation is consistent with recent data studying CHIKV infection in Vero cells using virus recombinant replicons [33]. In their paper, Fros et al. found that CHIKV infection efficiently blocked nuclear translocation of phosphorylated STAT1 in response to either type I or II IFNs. Other *Alphaviruses* like Semliki forest virus (SFV) and RRV were also reported to suppress the type I IFN response [34,35].

We cannot exclude the possibility that HT-1080 cells express only low levels of a functional receptor mediating CHIKV entry, yet to be characterized, but it should be stressed that the main differences in terms of susceptibility was not observed at early time point (8 h) but was evidenced at 24 h and 48 h. Counter-intuitively, the more resistant HT-1080 was producing lower levels of three main ISGs when compared to HS 633T cells at 48 h PI. We should further explore the possible contributions of other antiviral genes such as RNAse-L or PKR to explain differences between HS 633T and HT-1080 fibroblasts.

The analyses of the primary IFN and ISG responses to CHIKV infection did not explain the differences of susceptibility of the two fibroblast cell lines and experiments are now highly warranted to explore further possible additional mechanisms. We and others have recently shown that virus can hide into vesicles (blebs) to enter cells and escape classical recognition and antiviral mechanisms [8] which is likely to allow secondary infection of surrounding cells. It will be interesting to analyze the differential capacity of HT-1080 and HS 633T not only to generate and shed these blebs but also to engage macropinocytosis or phagocytosis through specific receptors.

## Conclusion

To sum up, the two human fibroblast cell lines HS 633T and HT-1080 represent good *in vitro* models to study CHIKV pathogenesis of the cognitive tissue given that they are differently susceptible to infection, replication and to engage an antiviral immune response. Gene profiling of the two cell lines should help to identify the different mechanisms involved in the response to CHIKV infection and subsequent infection of surrounding cells. The capacity of CHIKV to spread from cell to cell at later time point (>8 h) seems to be independent of the levels of RIG-I, TLR7, type I IFNs and ISGs. It will be essential to explore the contribution of novel cellular pathways such as macropinocytosis or phagocytosis to favor CHIKV infection of surrounding cells in spite of the innate immune response.

## Methods

### Cells and virus

Human fibrosarcoma cell lines, HS 633T and HT-1080 were obtained respectively from European Collection of Cell Cultures (ECACC, 89050201) and Pr Takashi Fujita (Tokyo, Japan). Cells were grown in Dulbecco’s Modified Eagle Medium (DMEM eagle, Sigma) supplemented with 10% fetal bovine serum (FBS) heat inactivated (Dutscher, P04-43100) and completed with 2 mM L-glutamine (Dutscher, P04-80100), 100U/mL - 0.1 mg/mL penicillin - streptomycin (Dutscher, P06-07100), 0.5 μg/mL fungizone (Dutscher, P06-01001), 1 mM sodium pyruvate (Dutscher, P04-43100). HS 633T cells were maintained at 37°C in a humid atmosphere with 5% CO_2_ in Petri dishes. We used a clinical isolate (CHIKV clone #4) amplified from a patient’s serum sample (isolated in our safety level 3 laboratory during the 2006 epidemic) by a single passage on Vero cells [32].

### Infection protocol

All infections were performed with CHIKV clone #4 at a MOI of 1. First, to test the susceptibility of the fibroblast cell lines HS 633T and HT-1080, cells were either grown on glass coverslips in 24-well plates or in Petri dishes until 60% of confluence and then incubated with CHIKV for different periods (8, 24 and 48 h PI). Mock-infected cells were prepared as a control. To compare the expression profile of HS 633T and HT-1080 innate immune genes, cells were grown in Petri dishes and infected with CHIKV for the same periods as above. Finally, to analyze the nuclear translocation of pSTAT1 in HS 633T and HT-1080, cells were grown on glass coverslips in 24-well plates until 40% of confluence and were either incubated with recombinant human IFN-α alone (Peprotech, 300-02A, France) for 30 min at a final concentration of 100 ng/mL, infected with CHIKV for 24 h and unstimulated or infected with CHIKV for 24 h and then incubated with human IFN-α for 30 min at a final concentration of 100 ng/mL.

### Immunofluorescence

Cells were washed twice in PBS, fixed for 5 min in cold absolute ethanol, dried for 10 min and conserved at −20°C. Cells were permeabilized with cold acetone for 30 sec, washed with PBS and blocked with 1% BSA in PBS. Coverslips were incubated at 4°C overnight in primary antibodies. To evaluate the susceptibility to CHIKV infection of HS 633T and HT-1080, a single immunostaining was performed with a human specific CHIKV antiserum (FDO, 1:4000 dilution). To determine if the nuclear translocation of pSTAT1 in infected HS 633T cells, a double immunostaining was performed with the following primary antibodies: monoclonal mouse anti-CHIKV (1:1000 dilution, as described [32] to detect the E1 envelope glycoprotein (a kind gift from Biomérieux, Marcy l’Etoile, France) or CHIKV antiserum FDO (1:4000 dilution) and polyclonal rabbit anti-human phosphoSTAT1 (1:200 dilution) (Millipore-Chemicon, 07–307). After washings, coverslips were incubated at room temperature for 2 h in secondary antibodies diluted at 1:1000. Goat anti-human Alexa Fluor 488 IgG (H + L), goat anti-mouse Alexa Fluor 488 IgG (H + L) and donkey anti-rabbit Alexa Fluor 594 IgG (H + L) (Invitrogen Molecular Probes) were used as secondary antibodies. Nuclei were stained with DAPI (Sigma, D9542) at a final concentration of 0.1 ng/mL. Coverslips were mounted in Vectashield (Vector Labs; Cliniscience), and fluorescence was observed using a Nikon Eclips 80i microscope. Images were obtained using the Nikon Digital camera system (Nikon, DXM1200C) and the imaging software NIS-Element BR version 3.1 (Nikon). Cells positive for pSTAT1 were counted in three random fields at 40X to evaluate the inhibition of pSTAT1 nuclear translocation in either HS 633T or HT-1080 mock-infected and unstimulated; mock-infected and IFN-α stimulated; CHIKV-infected and unstimulated; CHIKV-infected and IFN-α stimulated.

### Quantitative real-time RT-PCR (CHIKV E1 gene)

Supernatants were sampled from HS 633T or HT-1080 cell cultures and lysis buffer (NucliSENS® easyMAG®, 280134) was added v/v in a final volume of 1 mL. Total RNA was extracted from 200μL of supernatants in the easyMAG machine (BIOMERIEUX), according to the manufacturer protocol. A one step qRT-PCR was performed in a final volume of 20μL containing 2.5μL of extracted RNA, 10μL of 2X SuperScript® III Platinum® buffer (invitrogen), 10 μM of CHIKV E1 primers (Forward primer: AAG CTY CGC GTC CTT TAC CAA G, Reverse primer: CCA AAT TGT CCY GGT CTT CCT), 5 μM of probe (6 Fam-CCA ATG TCY TCM GCC TGG ACA CCT TT-Tamra), 40U/μL of RNAse Inhibitor (RNAsin, N2511, Promega), 1U/μL of uracil-DNA-glycosylase (UDG, 03539806001, Roche®), and 0.8μL of SuperScript® III RT/Platinum® *Taq* Enzyme Mix (invitrogren). qRT-PCRs was carried out in the LightCycler machine (version 1.5, Roche®) with the following steps: RT at 50°C for 20 min, PCR for pre-denaturation at 95°C for 2 min, 45 cycles of denaturation at 95°C for 5 sec and annealing at 60 °C for 1 min.

### Quantitative real time RT-PCR for innate immune genes

Total RNA was extracted with Trizol® Reagent (Life Technologies, Cat # 15596–026). qRT-PCR experiments were done either using a one step qRT-PCR procedure with TaqMan probes or a two step qRT-PCR assay in presence of a DNA-binding dye. All experiments were monitored on a LightCycler® 480 Instrument (Roche Diagnostics Ltd). The expression of RIG-I, ISG20, ISG54 and ISG56 (see Table 1) was assessed by a one-step quantitative RT-PCR performed using the One Step PrimeScript™ RT-PCR kit from TAKARA (Cat #RR064A, V.0701). Expression of IFN-β and TLR7 (see Table 1) was assessed by RT followed by a quantitative PCR using BRYT Green ® (Promega, Cat #A6001/2). In both procedures GAPDH was used as a housekeeping gene against which the gene relative expression was determined according to Pfaffl method [36]. All experiments were done in triplicate and relative expression levels were ploted with the software SigmaPlot version 12.0. Negative controls were included and PCR efficiency was determined from the slope of a dilution curve. 

**Table 1:** List of primers for quantitative RT-PCR

**Reference**	**Gene**	**Primer**	**5' modification**	**Sequence (5' to 3')**	**3' modification**
*Primer and probes used for the one-step qRT-PCR from TAKARA*					
NM_002201.4	**ISG20**	ISG20-636 F		CTGTTGTGGCGTGAGGCCA	
		ISG20-764R		TGCCCTCGCATCTTCCACC	
		ISG20-688P	HEX	TGCTGAGTGAGCGCCTCCTGC	BHQ-1
NM_002201.4	**ISG54**	ISG54-76 F		GGTCTCTTCAGCATTTATTGGTG	
		ISG54-219R		TGCCGTAGGCTGCTCTCCA	
		ISG54-124P	FAM	TGCAGCTGCCTGAACCGAGC	BHQ-1
NM_001548.3	**ISG56**	ISG56-131 F		GCCTAATTTACAGCAACCATGAG	
		ISG56-347R		GGCCTTTCAGGTGTTTCACATA	
		ISG56-215P	HEX	TGGGAGTTATCCATTGATGACGATG	BHQ-1
NM_014314.3	**RIG-I**	RIG-I-237 F		AGCTACATGGCCCCCTGGT	
		RIG-I-397R		GCATGGTCTAGGGCATCCAA	
		RIG-I-351P	HEX	CAGGAGGAAGGCTGGTTCCGT	BHQ-1
NM_002046	**GAPDH**	GAPDH-F		AGCCTCAAGATCATCAGCAATG	
		GAPDH-R		CTGTGGTCATGAGTCCTTCCA	
		GAPDH-P	HEX	CCAACTGCTTAGCACCCCTGGC	BHQ1
*Primers used for the RT-qPCR with BRYT Green*					
NM_002176.2	**IFN-β**	F	GTTCGTGTTGTCAACATGACCA		
		R	TCAATTGCCACAGGAGCTTCT		
NM_016562.3	**TLR7**	F	CCACAACCAACTGACCACTG		
		R	CCACCAGACAAACCACACAG		
NM_002046.3	**GAPDH**	F	GCACCGTCAAGGCTGAGAAC		
		R	GCCTTCTCCATGGTGGTGAA		

### Statistics

Percentages of infected HS 633T or HT-1080 cells, relative gene expression levels and percentages of HS 633T or HT-1080 cells with nuclear pSTAT1 were expressed as mean ± standard error (SEM) of 3 independent experiments, each using triplicate culture plates. Comparisons between all treatments (CTL, 8, 24, 48 h PI) for HS 633T or HT-1080 have been analyzed by a One-way ANOVA test. Values of p < 0.05 were considered statistically significant for a post-hoc Tukey-Kramer test in order to compare infected versus control samples. Comparison of gene expression between HS 633T and HT-1080 for each treatment mentioned above has been analyzed by a Two-way ANOVA test. Values of p < 0.05 were considered statistically significant to perform a Bonferroni post-hoc test. Inhibition of nuclear pSTAT1 translocation in both cell lines has been analyzed with a Student unpaired *t* test. All statistical tests were done using GraphPad Prism version 5.01. Degrees of significance are indicated in the figure caption as follow: * p < 0.05; ** p < 0.01; *** p < 0.001.

## Competing interests

The authors declare that they have no competing interests.

## Authors' contributions

PG designed research and secured financial support; VGTH, CG and GLPY performed research; PG, VGTH, MD, MCJB and CG analysed data; VGTH, MD and PG wrote the paper. All authors read and approved the submitted manuscript.
